# Effect of Different Soils on Pheromone-Enhanced Movement of Entomopathogenic Nematodes

**DOI:** 10.2478/jofnem-2025-0009

**Published:** 2025-03-29

**Authors:** Sehrish Gulzar, Kyle Slusher, Fatma Kaplan, Edwin E. Lewis, Steven Hobbs, David Shapiro-Ilan

**Affiliations:** USDA-ARS, SEA- SE Fruit and Tree Nut Research Unit, Byron, GA; Texas A&M, AgriLife Research and Extension Center Stephenville, TX; Pheronym, Inc. Woodland, CA; Department of Entomology, Plant Pathology and Nematology, University of Idaho, Moscow, ID

**Keywords:** Biocontrol efficacy, different soils, entomopathogenic nematodes, pheromones

## Abstract

Entomopathogenic nematodes (EPNs) have a specialized infective juvenile stage (IJ) that is mobile and has the capability to seek insect hosts to penetrate their haemocoel. EPNs are primarily applied to soil as biological control agents; thus, the IJs must move through the soil to find and infect a host. Soil characteristics are known to be an important factor that can affect the efficiency of EPN movement behavior. Previous research has shown that exposure to ascaroside pheromones can enhance EPN movement and infectivity in soil. The ability of pheromones to enhance EPN efficacy was recently demonstrated under field conditions in a pecan orchard. However, prior to our research, it was unknown whether different soils have differential effects on pheromone enhanced EPN efficacy. In different soils, we tested the biocontrol efficacy of *Steinernema carpocapsae*, *Steinernema feltiae* and *Heterorhabditis bacteriophora* in soil columns with and without pheromone exposure. All nematodes were evaluated in separate columns filled with oven dried commercial play sand and two different soils from pecan orchards (from Byron, GA and Tifton, GA). The soils differed substantially in several aspects such as field capacity, organic matter, nutrients, and nematode movement capacity. Efficacy was determined by baiting the bottom section of each column with larvae of the yellow mealworm (*Tenebrio molitor* L.). Results indicated that pheromones enhanced EPN efficacy for all EPN species and soils tested compared to treatments without pheromones. The magnitude/extent that pheromones boosted EPN movement in all EPNs regardless of soil type did not differ. Soil did not affect EPN efficacy for *H. bacteriophora* but did affect *S. carpocapsae* and *S. feltiae*. For both *S. carpocapsae* and *S. feltiae* efficacy was highest in the sandy field soil (Tifton soil) followed by that of the loamy sand (Byron soil) and pure sand (commercial play sand). When comparing the efficacy of EPN species to each other, we observed that *H. bacteriophora* killed more bait insects exposed to soil in the bottom of the soil column than other EPNs. Our findings suggest that pheromones can be used to enhance EPN efficacy in diverse soils. Future research may explore pheromone effects on EPNs in additional substrates.

Dispersal, host-finding, and infectivity are principal behaviors for parasites. A combination of extrinsic cues and intrinsic chemical drivers allied with potential hosts directs dispersal, foraging behavior, and infection (Kaplan et al., 2020). Local environmental conditions also affect a parasite’s success. For example, for soil-inhabiting parasites environmental factors impacting dispersal include soil moisture, soil type, temperature, salinity, etc. (Kaspi et al., 2010).

Entomopathogenic nematodes (EPNs) belonging to the genera *Steinernema* and *Heterorhabditis* are biological control agents that are used to control a broad range of economically important insect pests (Shapiro-Ilan et al., 2017, 2018; Shapiro-Ilan and Lewis, 2024). These nematodes naturally inhabit soil and kill their hosts with the aid of symbiotic bacteria: *steinernematids* are associated with *Xenorhabdus* spp. and *heterorhabditids* are associated with *Photorhabdus* spp. bacteria. Once nematodes consume an insect host, a specialized, non-developmental stage called the infective juvenile (IJ) develops inside the spent host, which disperses and forages to search for a new host. The IJs typically move through the soil environment to find their next host.

EPN foraging strategies have been categorized on a continuum from ambushing to cruising behavior (Campbell et al., 1998; Lewis et al., 2006; Griffin, 2012; Lewis et al., 2024). While in the soil or on the soil surface, ambushers perform a sit and wait strategy and spend a considerable amount of host-finding time in a stationary position, often standing on their tails and waiting for a host to pass by. *Steinernema carpocapsae* has been classified as an example of an ambusher (Lewis et al., 1992). Cruising nematodes are more active in moving in the soil and searching for a host to infect, they generally do not tail stand or jump (as ambushers do). For example, *Steinernema glaseri* and *Heterorhabditis* spp. are classified as cruisers. Intermediate foragers often exhibit foraging behaviors that entail characteristics of both ambushers and cruisers; *Steinernema feltiae* is, for example, considered to be an intermediate forager (Selvan et al., 1993; Campbell and Kaya, 2002; Lewis et al., 2006).

EPN movement in different soils has been observed to vary. In heavier soils, infectivity and movement are hindered, whereas in light loam soils with similar percentages of silt and clay, their movement is facilitated (Brzeski and Sandner, 1974; Kaya, 1990; Hazir et al., 2003; Campos-Herrera and Gutierrez, 2009; Shapiro-Ilan et al., 2009). Infectivity of many EPNs increases with soil sand content (Kung et al., 1990; Portillo-Aguilar et al., 1999). IJs penetrate hosts more quickly in sandy and sandy loam soils (Kaya, 1990). Kaspi et al. (2010) observed the highest nematode foraging efficacy and infectivity in sandy soils. Soil with the appropriate moisture levels also facilitates EPN movement and host-finding. The movement of nematodes within soil pores is enabled by a thin water capsule around the body of the nematode (Norton, 1978; Kaya and Gaugler, 1993; Shapiro-Ilan and Gaugler, 2002; Glazer, 2002). Soil pH is a key factor in EPN activity, acidic soil pH levels cause decreases in IJ infectivity and survival. The optimal pH range of soil for the growth and reproduction of most plant species and soil organisms is between 5.5 and 7.2 (Stuczynski et al., 2004).

Given that foraging and infectivity are critical to EPN success, factors that impact foraging and infectivity in soil can lead to improved efficacy. EPN ascaroside pheromones can increase EPN movement and infectivity in soil (Oliveira-Hofman et al., 2019; Shapiro et al., 2019). Thus, pheromones may be used to boost the efficacy of EPNs in pecan systems and in other commodities. Examples of improved EPN efficacy based on pheromones thus far only include sand in laboratory and greenhouse studies (Oliveira-Hofman et al., 2019; Shapiro et al., 2019) and one field experiment in a pecan orchard targeting pecan weevil (Perier et al., 2024). However, the impact of different soils on pheromone activity has not been studied. In this study, we tested the effect of different soils on the pheromone booster effect. We tested the efficacy of EPNs with three different foraging strategies with or without pheromone pre-treatment in three different soils. We chose pure sand (commercial play sand (as a baseline) and two soils from pecan orchards, a sandy field soil (Tifton) and loamy sand (Byron). The soils differed substantially in several aspects such as field capacity, pH, organic matter, nutrients, and nematode movement capacity. For the purposes of this paper, we define efficacy as relative measure of the ability to move through the soil, infect and kill insect pests.

## Materials and Methods

### Insect source and nematode culture

*Galleria mellonella* (L.) (Lepidoptera: Pyralidae) and *Tenebrio molitor* L. (Coleoptera: Tenebrionidae) used in this experiment were obtained from Southeastern insectaries, Inc. (Perry, GA).

All nematode strains were cultured *in vivo* at 25°C in the last instars of commercially obtained *G. mellonella* based on methods described by Hazir et al. (2022). Briefly, IJs were collected from cadavers using a White trap (White, 1927). This method involved placing *G. mellonella* larvae that were previously infected with EPNs into a 60 × 15 mm Petri dish lined with moistened filter paper. This Petri dish was placed inside of a 90 × 15 mm Petri dish filled with 30–40 ml of water which the IJs would migrate to upon emergence. IJs were collected from the water every day to prevent high mortality due to lack of oxygen. All of the emerged IJs were stored at 14 °C until use (no later than two weeks post-harvest).

### Deconditioning the nematodes

Prior to experimentation, all nematodes went through a sensitization process to remove any residual pheromones from the *in vivo* cultures. To optimally detect a pheromone response, nematodes need to be sensitized to pheromones by washing followed by removing excess water from them and stored for a period of 11–14 days (Srinivasan et al., 2008; Kaplan et al., 2011, 2012; Oliveira-Hofman et al., 2019; Shapiro-Il et al., 2019). EPN washing was done by vacuum filtration. Excess water was removed into a plastic jar (below the filter) and all IJs remained on the top of the filter. IJs were rinsed three times with deionized water. After the final wash, deconditioned EPNs were resuspended in deionized water and stored in a culture flask at 14 °C for 11–14 days prior to the experiment to ensure that the IJs were deconditioned.

### Pheromone extract

*S. feltiae* pheromone extracts were obtained as described by Kaplan et al. (2020). Briefly, pheromones were extracted using 70% methyl alcohol from *S. feltiae* consumed host cadavers (Kaplan et al., 2012; Oliveira-Hofman et al., 2019; Shapiro-Il et al., 2019). Infected cadavers were harvested and used within 10 days of initial IJ emergence. The cadavers were mixed with 70% methanol (one cadaver in 1 ml of 70% methyl alcohol) in an incubator shaker (New Brunswick Scientific, Ontario, CA) at 150 rpm at room temperature for 10 min. The supernatant was collected by centrifugation at 10000 rcf for 15 min and dried in a rotary evaporator. The extract was then resuspended in 10X concentration using purified water (ELGA Purelab Ultra, High Wycombe, UK) and centrifuged at 6000g for 15 min. The supernatant was lyophilized in a 4-L 8 manifold lyophilizer and then stored in −80 °C.

### Efficacy bioassay

Efficacy was measured for *S. carpocapsae* (All strain), *S. feltiae* (SN) and *H. bacteriophora* (VS) in columns made of three stacked sections (4 cm diameter × 5.3 cm length per section and full length of 16cm) of polyvinyl chloride (PVC) pipes as described by Wu et al. (2018), Oliveira-Hofman et al. (2019), and Slusher et al. (2024). The three EPN species were tested in separate columns. Three different soils were tested: commercial play sand (Quikrete® Premium Play 76 Sand, Atlanta, USA), and soil obtained from two different pecan orchards, one in Tifton, GA (University of Georgia Ponder Research Farm (31°30′29.1″N 83°38′16.0″W)) and one in Byron, GA (USDA-ARS Southeastern Fruit and Tree Nut Research Station (32°39′30.9″N 83°44′31.4″W)). Soil characteristics are provided in Table 1. The soils differed substantially in several characteristics including organic matter, field capacity and nutrients. Each column was separately filled with soil from one of the three different soils and maintained at their corresponding field capacity (field capacities were 8%, 13.5% and 14.5% for commercial play sand, Tifton orchard soil, and Byron orchard soil, respectively). Approximately, 3,500 IJs of each EPN species were added to the top of each vertically placed column. EPNs were introduced in either a suspension with pheromones or without (control). For the pheromone treatment, resuspended 100 host cadaver equivalent (HCE) Sf pheromone extracts (Kaplan et al., 2012) in 10 ml of deionized water were added to ~100,000 pheromones deconditioned IJs in 10 ml of water to a pheromone mixture (10ml) (this made the final pheromone concentration 1X). The total volume was 20 ml (pheromone +IJs) and was then incubated for 20 min by placing the suspension on its side on a rocking table to ensure aeration. For non-pheromone treatments, IJs were resuspended in deionized water at the same volume. The tops of the soil columns were covered and stored in an incubator at 25 °C for three days to allow for the nematode movement.

**Table 1:** Characteristics of three soils included in entomopathogenic nematode movement studies.

	**Tifton Orchard soil**	**Byron Orchard soil**	**Play Sand**
Field-capacity (%)	13.5	14.5	8
Low cation buffer (LCB) (ppm CaCO_3_/Ph)	238.0	353.0	49.00
pH CaCl_2_	5.48	4.77	6.16
Equivalent water pH	6.08	5.37	6.76
Sand (%)	98.1	84.1	100.0
Silt (%)	1.9	13.6	0.0
Clay (%)	0.0	2.2	0.0
Organic matter (%)	1.670	6.259	0.0340
Calcium (kg/ha)	1217.24	2252.91	178.77
Potassium (kg/ha)	132.82	219.68	57.42
Magnesium (kg/ha)	181.12	278.97	17.86
Manganese (kg/ha)	15.13	299.26	2.953
Phosphorus (kg/ha)	337.93	162.18	11.44
Zinc (kg/ha)	32.78	51.98	1.769

Treatment comparisons were made by assessing EPN efficacy of IJs that moved into the bottom section of the columns (Wu et al., 2018; Oliveira-Hofman et al., 2019). To test for movement and infectivity, soil from the bottom section of each PVC column was placed into Petri dishes and baited with 10 *T. molitor* larvae. After three days of incubation at 25 °C, the number of dead insects was determined. If 100% mortality was achieved, the plates were rebaited (three-day incubation) until less than 100% mortality was observed. Total insect mortality from the multiple baitings was compared among treatments.

There were three replicates for each nematode*soil* pheromone combination and the entire experiment was repeated in time.

### Statistical analysis

Data were analyzed using a generalized linear model (GLM) followed by Tukey’s HSD test (SAS, 2011). To determine the effect of pheromone treatment within each soil type, each EPN species was run separately. Factorial analysis was applied with pheromone and soil as main factors. There was no significant interaction between soil type and pheromone, so we were able to look at differences across the main variables. Subsequently, to compare the relative pheromone-boosting effect (pheromone improvement) among the EPN species and soils, we calculated the ratio of dead bait insects in the pheromone treatment columns relative to (divided by) the corresponding number of insects killed in the non-pheromone control columns for each species; this calculation allowed us to compare the magnitude of pheromone-boosting effects among nematodes and among the soils. No significant interaction between main variables was observed so again only main effects are reported. Lastly, we compared the efficacy among EPN species. Data transformations were not applied based on residual plots.

## Results

**Figure 1:**
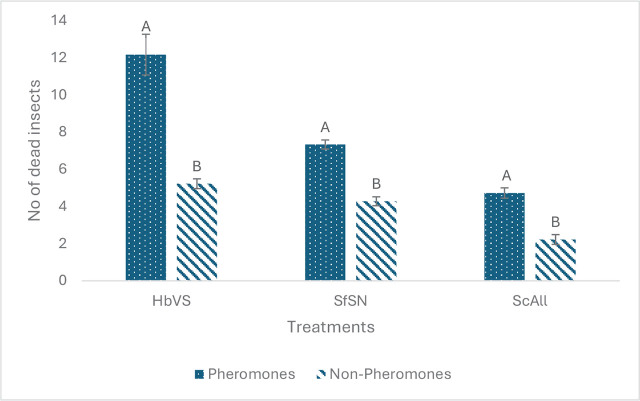
Mean (± SE) number of dead *Tenebrio molitor* larvae resulting from exposure to *Heterorhabditis bacteriophora* (HbVS), *Steinernema feltiae* (SfSN) and *Steinernema carpocapsae* (ScAll). The nematodes were either exposed to pheromones or not exposed. Insect baiting was done in different soils (the soil effects are combined across treatments): commercial play sand, Byron orchard soil (B) and Tifton orchard soil (T). The number of dead insects reflect the level of nematode efficacy after moving through a soil column; The soil from the bottom section of a soil column was exposed to the insects. Bars with the same letter are not significantly different within each nematode species (Tukey’s test, alpha = 0.05).

There was no significant interaction between soil and pheromones (*p* = 0.7309 for *H. bacteriophora*, *p* = 0.8984 for *S. carpocapsae*, *p* = 0.6812 for *S. feltiae*). Results indicated that pheromones enhanced EPN efficacy for all the EPN species and soils relative to non-pheromone controls (*F* = 33.45, df = 1, 29, *p* <0.0001 for *H. bacteriophora*, *F* = 72.59, df = 1, 29, *p* <0.0001 for *S. carpocapsae*, *F* = 90.53, df = 1, 29, *p* <0.0001 for *S. feltiae*) (Fig. 1).

**Figure 2:**
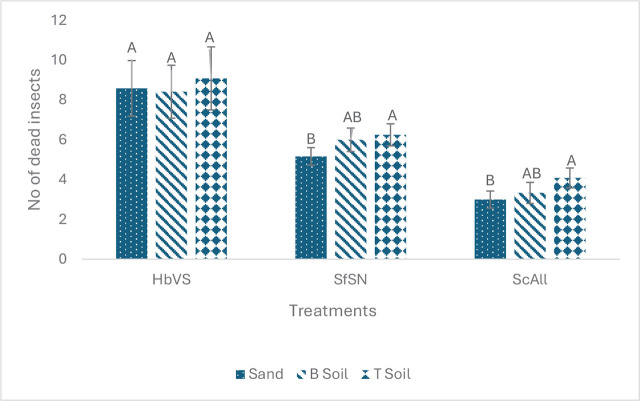
Mean (± SE) number of dead *Tenebrio molitor* larvae resulting from exposure to *Heterorhabditis bacteriophora* (HbVS), *Steinernema feltiae* (SfSN) and *Steinernema carpocapsae* (ScAll) baiting in different soils: commercial play sand, Byron orchard soil (B) and Tifton orchard soil (T). The number of dead insects reflect the level of nematode efficacy after moving through a soil column; The soil from the bottom section of a soil column was exposed to the insects. Bars with the same letter within species are not significantly different (Tukey’s test, alpha = 0.05).

Next, we tested whether soil types influenced EPN efficacy without pheromone treatment. Soil did not affect EPN efficacy of *H. bacteriophora* (*F* = 0.11, df = 2, 29, *p* = 0.895). However, soils affected efficacy of the *S. carpocapsae* (*F* = 4.77, df = 2, 29, *p* 0.0162), and *S. feltiae* (*F* = 4.16, df = 2, 29, *p* = 0.0258) (Fig. 2). Both *S. carpocapsae* and *S. feltiae* efficacy was higher in the Tifton soil followed by the Byron pecan soil (intermediate) and the commercial play sand.

**Figure 3:**
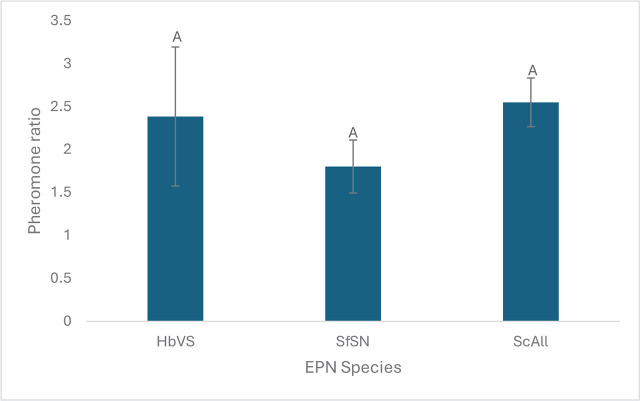
Mean (± SE) ratios of dead *Tenebrio molitor* in soil baited with *Heterorhabditis bacteriophora*, *Steinernema feltiae* and *Steinernema carpocapsae* exposed to pheromone (treatment) divided by dead insects from nematodes not exposed to pheromones (control). The number of dead insects reflect the level of nematode efficacy after moving through a soil column; The soil from the bottom section of a soil column was exposed to the insects. This ratio measures the magnitude of the pheromone boosting effect on nematode efficacy among species. Bars with the same letter are not significantly different (Tukey’s test, alpha = 0.05).

**Figure 4:**
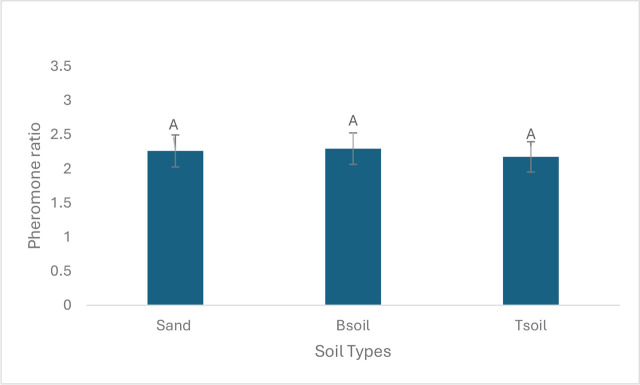
Mean (± SE) ratios of dead *Tenebrio molitor* in different soils baited with entomopathogenic nematodes (*Heterorhabditis bacteriophora*, *Steinernema feltiae* and *Steinernema carpocapsae*) exposed to pheromone (treatment) divided by dead insects from nematodes not exposed to pheromones (control). Nematode species effects are combined across treatments. The ratio measures the magnitude of the relative pheromone boosting effect on different soils. The number of dead insects reflect the level of nematode efficacy after moving through a soil column; The soil from the bottom section of a soil column was exposed to the insects. Bars with the same letter are not significantly different (Tukey’s test, alpha = 0.05).

In testing the magnitude of the *S. feltiae* pheromone-booster effect, no significant interaction between the main factors (soil and pheromone) was detected (*p* = 0.5039). We found no significant differences in the magnitude of the *S. feltiae* pheromone effect on the three EPN species (*F* = 2.98, df = 2, 44, *p* = 0.061) (Fig. 3). Similarly, no significant differences in magnitude of pheromone effect were observed among soils (*F* = 0.1, df = 2, 44, *p* = 0.902) (Fig. 4). This indicates that the magnitude/extent that the pheromone enhances the EPN efficacy did not differ based on EPN species or soil types.

**Figure 5:**
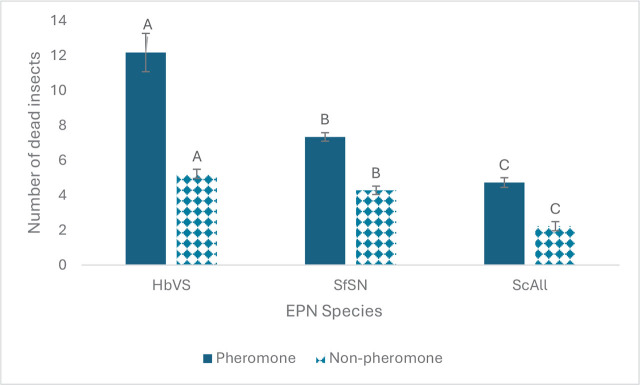
Mean (± SE) number of dead *Tenebrio molitor* larvae resulting from *Heterorhabditis bacteriophora* (HbVS), *Steinernema feltiae* (SfSN) and *Steinernema carpocapsae* (ScAll) with and without pheromone exposure in different soils: commercial play sand, Byron orchard soil (B) and Tifton orchard soil(T) (the soil effects are combined across treatments). The number of dead insects reflect the level of nematode efficacy after moving through a soil column; The soil from the bottom section of a soil column was exposed to the insects. Bars with the same letter within each pheromone series are not significantly different (Tukey’s test, alpha = 0.05).

In comparing overall movement among EPN species, there was no significant interaction between soil and EPN species (*p* = 0.9462). However, there was a significant interaction observed between the *S. feltiae* pheromone and EPN species (*p* <.0001). So, we separated the analysis for each level of pheromones (both those with and without) (Fig. 5). Efficacy of all the EPN species were significantly different from each other (*F* = 52.1, df = 2, 95, *p* = <.0001). For both pheromones exposed to EPNs and non-treated EPNs, *H. bacteriophora* had higher efficacy compared to *S. feltiae*, which was in turn higher than *S. carpocapsae*

## Discussion

Our results indicate that pheromones enhanced efficacies for all EPN species and in all soils, relative to no pheromone controls. Previous research has shown that ascaroside pheromones can enhance the dispersal of *steinernematid* and *heterorhabditid* EPNs (Oliveira-Hofman et al., 2019; Kaplan et al., 2012, 2020; Wang et al., 2022). Ascaroside pheromones were shown to enhance EPN movement in soil (Oliveira-Hofman et al., 2019) and enhance infectivity (Shapiro-Ilan et al., 2019). However, prior to our research it was unknown whether the pheromone booster effect would differ among different soils. Results indicate that there was no difference in magnitude/extent of pheromone effect on EPN species and soils. So based on the results we expect that pheromones can be used across different soils and with the EPNs tested to boost EPN efficacy.

EPNs were differentially impacted by the different soils. *H. bacteriophora* appeared to move equally well in all the soils. In contrast, *S. carpocapsae* and *S. feltiae* efficacy was higher in the Tifton soil than the commercial play sand and movement in the Byron pecan soil was intermediate. EPNs generally move most efficiently when their body length is three times the average diameter of soil particles (Nicholas, 1984). Many studies have shown that EPN foraging efficacy is higher in sandy soil than heavier soils (Georgis and Poinar, 1983; Kaspi et al., 2010) and that infectivity of many EPNs increases with soil sand content (Molyneux and Bedding, 1984; Geden et al., 1985; Kung et al., 1990; Portillo-Aguilar et al., 1999; Koppenhöfer and Fuzy, 2006). Our results did not support prior studies in which sandier soils were more conducive to EPN efficacy as the Byron soil and Tifton soil did not differ for any of the EPN species. However, other studies have observed exceptions as well (Shapiro et al., 2000). It is not clear why the Tifton soil had greater movement than plain commercial sand. The higher organic matter in the Tifton soil may have led to less compaction and thus greater nematode movement. Soil pH can also affect nematode efficacy, with certain nematode species preferring more acidic or alkaline conditions. For example, *S. feltiae* and *H. bacteriophora* showed greater mobility, activity, and pathogenicity at pH 6.8 and 8 than at pH 5.5 (Jaworska and Dudek, 1992). Khathwayo et al. (2021) observed reduced survival of certain EPN species at a pH of 5 and below. Thus, the low pH of the Byron soil and Tifton soils may have impacted EPN activity.

In the overall movement comparison among EPN species, *H. bacteriophora* moved more than *S. feltiae* and *S. carpocapsae.* These results are consistent with EPN foraging strategies, *H. bacteriophora* acts as a cruiser, which tends to move more actively than ambusher nematodes such as *S. carpocapsae* (and *S. feltiae* is intermediate). Thus, more *H. bacteriophora* may have moved to the bottom section relative to *S. carpocapsae* (with *S. feltiae* being intermediate) due to their foraging behavior. Prior research also reported cruiser nematodes and or intermediate EPN species moving farther down a soil column compared to an ambusher (e.g., *S. carpocapsae*) (Grewal et al, 1994; Slusher et al., 2024).

Various studies have highlighted the use of pheromones as boosters to improve the efficacy of EPNs (Wu et al., 2018; Shapiro-Ilan et al., 2019; Kaplan et al., 2020). Pheromones have also been shown to stimulate dispersal under cool temperatures (Kaplan et al., 2020). Other studies have also observed conspecific and heterospecific pheromone effects (Kaplan et al., 2012, Shapiro-Ilan et al., 2019; Kaplan et al., 2020). Our results expand the utility of pheromone use in that we have demonstrated that the booster effect is observed across soils.

Additional studies are needed to explore the usefulness of EPN pheromones under varying conditions. For example, future studies should focus on assessing the efficacy of EPN pheromones in a wider array of soil types, soil conditions, and other media (such as in wood galleries). Moreover, in this study, we investigated the impact of different soils on the overall efficacy of EPNs encompassing both dispersal and infectivity; future research can investigate whether dispersal and infectivity behaviors are impacted differently. Research is also needed to test the efficacy of pheromone boosters in a wider variety of pests and cropping systems. Other booster compounds have been discovered. For example, 3-methyl-3-buten-1-ol, was found to be an effective cue for EPN attraction and infection (Mbata et al., 2019). Additionally, thiourea was found to be a very promising EPN booster, as the compound suppresses the host immune system and can thereby increase their virulence (Li et al., 2024). Future studies may include testing pheromones in combination with other boosters (e.g., 3-methyl-3-buten-1-ol or thiourea) for EPN efficacy in different types of soils.

## References

[j_jofnem-2025-0009_ref_001] Brzeski M, Sandner H (1974). Zar ys nematologii.

[j_jofnem-2025-0009_ref_002] Campbell JF, Kaya HK (2002). Variation in entomopathogenic nematode (Steinernematidae and Heterorhabditidae) infective stage jumping behavior. Nematology.

[j_jofnem-2025-0009_ref_003] Campbell JF, Orza G, Yoder F, Lewis E, Gaugler R (1998). Spatial and temporal distribution of endemic and released entomopathogenic nematode populations in turfgrass. Entomologia Experimentalis et Applicata.

[j_jofnem-2025-0009_ref_004] Campos-Herrera R, Gutierrez C (2009). Screening Spanish isolates of Steinernematid nematodes for use as biological control agents through laboratory and greenhouse microcosm studies. Journal of Invertebrate Pathology.

[j_jofnem-2025-0009_ref_005] Geden CJ, Axtell RC, Brooks WM (1985). Susceptibility of the lesser mealworm *Alphitobius diaperinus* (Coleoptera: Tenebrionidae) to the entomogenous nematodes *Steinernema feltiae, S. glaseri* (Steinernematidae) and *Heterorhabditis heliothidis* (Heterorhabditidae). Journal of Entomological Science.

[j_jofnem-2025-0009_ref_006] Georgis R, Poinar GO (1983). Vertical migration of *Heterorhabditis bacteriophora* and *H. heliothidis* (Nematoda: Heterorhabditidae) in sandy loam soil. Journal of Nematology.

[j_jofnem-2025-0009_ref_007] Glazer I, Gaugler R (2002). Survival biology. Entomopathogenic nematology.

[j_jofnem-2025-0009_ref_008] Grewal PS, Gaugler R, Campbell JF (1994). Host finding behaviour as a predictor of foraging strategy in entomopathogenic nematodes. Parasitology.

[j_jofnem-2025-0009_ref_009] Griffin CT (2012). Perspectives on the behavior of entomopathogenic nematodes from dispersal to reproduction: traits contributing to nematode fitness and biocontrol efficacy. Journal of Nematology.

[j_jofnem-2025-0009_ref_010] Hazir S, Kaya H, Touray M, Cimen H, Shapiro-Ilan D (2022). Basic laboratory and field manual for conducting research with the entomopathogenic nematodes, *Steinernema* and *Heterorhabditis*, and their bacterial symbionts. Turkish Journal of Zoology.

[j_jofnem-2025-0009_ref_011] Hazir S, Kaya HK, Stock S, Keskin N (2003). Entomopathogenic nematodes (Steinernematidae and Heterorhabditidae) for biological control of soil pests. Turkish Journal of Biology.

[j_jofnem-2025-0009_ref_012] Jaworska M, Dudek B (1992). Wyst. epowanie owadobójczych nicieni w glebach wybranych upraw. Zeszyty Naukowe Akademii Gorniczo-Hutniczej Kraków.

[j_jofnem-2025-0009_ref_013] Kaplan F, Alborn HT, von Reuss SH, Ajredini R, Ali JG, Akyazi F (2012). Interspecific nematode signals regulate dispersal behavior. PLoS One.

[j_jofnem-2025-0009_ref_014] Kaplan F, Perret-Gentil A, Giurintano J, Stevens G, Erdogan H, Schiller KC (2010). Conspecific and heterospecific pheromones stimulate dispersal of entomopathogenic nematodes during quiescence. Scientific Reports.

[j_jofnem-2025-0009_ref_015] Kaplan F, Srinivasan J, Mahanti P, Ajredini R, Durak O, Nimalendran R (2011). Ascaroside expression in Caenorhabditis elegans is strongly dependent on diet and developmental stage. PLoS One.

[j_jofnem-2025-0009_ref_016] Kaspi R, Ross A, Hodson AK, Stevens GN, Kaya HK, Lewis EE (2010). Foraging efficacy of the entomopathogenic nematode *Steinernema riobrave* in different soil types from California citrus groves. Applied Soil Ecology.

[j_jofnem-2025-0009_ref_017] Kaya HK, Gaugler R, Kaya HK (1990). Soil Ecology. Entomopathogenic nematodes in biological control.

[j_jofnem-2025-0009_ref_018] Kaya HK, Gaugler R (1993). Entomopathogenic nematodes. Annual Review of Entomology.

[j_jofnem-2025-0009_ref_019] Khathwayo Z, Ramakuwela T, Hatting J, Shapiro-Ilan DI, Cochrane N (2021). Quantification of pH tolerance levels among entomopathogenic nematodes. Journal of Nematology [Internet].

[j_jofnem-2025-0009_ref_020] Koppenhöfer AM, Fuzy EM (2006). Effect of soil type on infectivity and persistence of the entomopathogenic nematodes *Steinernema scarabaei, Steinernema glaseri, Heterorhabditis zealandica,* and *Heterorhabditis bacteriophora*. Journal of Invertebrate Pathology.

[j_jofnem-2025-0009_ref_021] Kung SP, Gaugler R, Kaya HK (1990). Soil type and entomopathogenic nematode persistence. Journal of Invertebrate Pathology.

[j_jofnem-2025-0009_ref_022] Lewis EE, Gaugler R, Harrison R (1992). Entomopathogenic nematode host finding: Response to host contact cues by cruise and ambush foragers. Parasitology.

[j_jofnem-2025-0009_ref_023] Lewis EE, Campbell J, Griffin C, Kaya H, Peters A (2006). Behavioral ecology of entomopathogenic nematodes. Biological Control.

[j_jofnem-2025-0009_ref_024] Lewis EE, Stevens G, Hiltpold I, Shapiro-Ilan DI, Shapiro-Ilan DI, Lewis EE (2024). Behavioral ecology of entomopathogenic nematodes. Entomopathogenic nematodes as biological control agents.

[j_jofnem-2025-0009_ref_025] Li X, Shapiro-Ilan DI, Tarasco E, Zeng S, Liu Q, Yang W (2024). Thiourea as a polyphenol oxidase inhibitor enhances host infection by the entomopathogenic nematode, *Heterorhabditis beicherriana*. Biological Control.

[j_jofnem-2025-0009_ref_026] Mbata GN, Shapiro-Ilan DI, Alborn HT, Strand MR (2019). Preferential infectivity of entomopathogenic nematodes in an envenomed host. International Journal for Parasitology.

[j_jofnem-2025-0009_ref_027] Molyneux AS, Bedding RA (1984). Influence of soil texture and moisture on the infectivity of *Heterorhabditis* sp. D1 and *Steinernema glaseri* for larvae of the sheep blowfly *Lucilia cuprina*. Nematologica.

[j_jofnem-2025-0009_ref_028] Nicholas WL (1984). The biology of free-living nematodes.

[j_jofnem-2025-0009_ref_029] Norton DC (1978). Ecology of plant parasitic nematodes.

[j_jofnem-2025-0009_ref_030] Oliveira-Hofman C, Kaplan F, Stevens G, Lewis EE, Wu S, Alborn HT (2019). Pheromone extracts act as boosters for entomopathogenic nematodes efficacy. Journal of Invertebrate Pathology.

[j_jofnem-2025-0009_ref_031] Perier JD, Kaplan F, Lewis EE, Alborn H, Schliekelman P, Toews MD (2024). Enhancing entomopathogenic nematode efficacy with pheromones: A field study targeting the pecan weevil. Journal of Invertebrate Pathology.

[j_jofnem-2025-0009_ref_032] Portillo-Aguilar C, Villani MG, Tauber MJ, Tauber CA, Nyrop JP (1999). Entomopathogenic nematode (Rhabditida: Heterorhabditidae and Steinernematidae) response to soil texture and bulk density. Environmental Entomology.

[j_jofnem-2025-0009_ref_033] Selvan S, Gaugler R, Lewis EE (1993). Biochemical energy reserves of entomopathogenic nematodes. Journal of Parasitology.

[j_jofnem-2025-0009_ref_034] Shapiro DI, Kaplan F, Oliveira-Hofman C, Schliekelman P, Alborn HT, Lewis EE (2019). Conspecific pheromone extracts enhance entomopathogenic infect. Journal of Nematology [Internet].

[j_jofnem-2025-0009_ref_035] Shapiro DI, Mccoy CW, Fares A, Obreza T, Dou H (2000). Effects of soil type on virulence and persistence of entomopathogenic nematodes in relation to control of *Diaprepes abbreviates* (Coleoptera: Curculionidae). Environmental Entomology.

[j_jofnem-2025-0009_ref_036] Shapiro-Ilan DI, Hazir S, Glazer I, Lacey LA (2017). Basic and applied research: entomopathogenic nematodes. Microbial agents for control of insect pests: From discovery to commercial development and use.

[j_jofnem-2025-0009_ref_037] Shapiro-Ilan DI, Hiltpold I, Lewis EE, Hajek AE, Shapiro-Ilan DI (2018). Ecology of invertebrate pathogens: Nematodes. Ecology of invertebrate diseases.

[j_jofnem-2025-0009_ref_038] Shapiro-Ilan DI, Lewis EE (2024). Entomopathogenic nematodes as biological control agents.

[j_jofnem-2025-0009_ref_039] Shapiro-Ilan DI, Gaugler R (2002). Production technology for entomopathogenic nematodes and their bacterial symbionts. Journal of Industrial Microbiology and Biotechnology.

[j_jofnem-2025-0009_ref_040] Shapiro-Ilan DI, Campbell JF, Lewis EE, Elkon JM, Kim-Shapiro DB (2009). Directional response of *Steinernematid* nematodes in response to electrical current. Journal of Inver tebrate Pathology.

[j_jofnem-2025-0009_ref_041] Slusher EK, Lewis E, Stevens G, Shapiro DI (2024). Movers and shakers: Do nematodes that move more invade more?. Journal of Invertebrate Pathology.

[j_jofnem-2025-0009_ref_042] Srinivasan J, Kaplan F, Ajredini R, Zachariah C, Alborn HT, Teal PE (2008). A blend of small molecules regulates both mating and development in *Caenorhabditis elegans*. Nature [Internet].

[j_jofnem-2025-0009_ref_043] Stuczynski T, Siebielec G, Maliszewska-Kordybach B, Smreczak B, Gawrysiak L (2004). Wyznaczanie obszarów, na których przekroczone sa. standardy jakości Gleb.

[j_jofnem-2025-0009_ref_044] Wang J, Cao L, Huang Z, Gu X, Cui Y, Li J (2022). Influence of the ascarosides on the recovery, yield and dispersal of entomopathogenic nematodes. Journal of Invertebrate Pathology [Internet].

[j_jofnem-2025-0009_ref_045] White GF (1927). A method for obtaining infective nematode larvae from cultures. Science.

[j_jofnem-2025-0009_ref_046] Wu S, Kaplan F, Lewis E, Alborn HT, Shapiro-Ilan DI (2018). Infected host macerate enhances entomopathogenic nematode movement towards hosts and infectivity in a soil profile. Journal of Invertebrate Pathology [Internet].

